# Omeprazole Administration in Preterm Preeclampsia: a Randomized Controlled Trial to Study Its Effect on sFlt-1 (Soluble Fms-Like Tyrosine Kinase-1), PlGF (Placental Growth Factor), and ET-1 (Endothelin-1)

**DOI:** 10.1161/HYPERTENSIONAHA.122.19070

**Published:** 2022-04-28

**Authors:** Rugina I. Neuman, Milan D. Baars, Langeza Saleh, Michelle Broekhuizen, Daan Nieboer, Jérôme Cornette, Sam Schoenmakers, Michel Verhoeven, Birgit C.P. Koch, Henk Russcher, Sjoerd A.A. van den Berg, Anton H. van den Meiracker, Willy Visser, A.H. Jan Danser

**Affiliations:** Department of Internal Medicine, Division of Pharmacology and Vascular Medicine (R.I.N., L.S., M.B., A.H.v.d.M., W.V., A.H.J.D.), Erasmus MC University Medical Center, Rotterdam, The Netherlands.; Department of Obstetrics and Gynecology, Division of Obstetrics and Fetal Medicine (R.I.N., M.D.B., L.S., J.C., S.S., W.V.), Erasmus MC University Medical Center, Rotterdam, The Netherlands.; Department of Pediatrics, Division of Neonatology (M.B.), Erasmus MC University Medical Center, Rotterdam, The Netherlands.; Department of Biochemical Statistics (D.N.), Erasmus MC University Medical Center, Rotterdam, The Netherlands.; Department of Pharmacy (M.V., B.C.P.K.), Erasmus MC University Medical Center, Rotterdam, The Netherlands.; Department of Clinical Chemistry (H.R., S.A.A.v.d.B.), Erasmus MC University Medical Center, Rotterdam, The Netherlands.; Department of Internal Medicine, Division of Endocrinology (S.A.A.v.d.B.), Erasmus MC University Medical Center, Rotterdam, The Netherlands.

**Keywords:** adult, endothelin-1, maternal age, perfusion, placenta

## Abstract

Low sFlt-1 (soluble Fms-like tyrosine kinase-1) and ET-1 (endothelin-1) levels have been reported in preeclamptic women using proton pump inhibitors.

**Methods:**

Here, we examined whether the proton pump inhibitor omeprazole could acutely reduce sFlt-1 and ET-1 (measured as CT-proET-1 [C-terminal pro-endothelin-1]), or increase free PlGF (placental growth factor) in 20 women with confirmed preeclampsia. Primary outcome was specified as the difference in sFlt-1, PlGF, or CT-proET-1 after 4 days of omeprazole versus 20 preeclamptic women not receiving omeprazole.

**Results:**

Mean maternal age was 30 years, and median gestational age was 30^+3^ weeks. Baseline sFlt-1 levels were identical in both groups, and the same was true for PlGF or CT-proET-1. After 4 days, sFlt-1 levels remained similar in women not receiving omeprazole compared with women receiving omeprazole, while the levels of PlGF and CT-proET-1 also did not differ between groups. Women receiving omeprazole had a similar prolongation of pregnancy after inclusion compared with those in the nonomeprazole group (median 15 versus 14 days). Except for a higher neonatal intubation rate in the nonomeprazole group (31% versus 4%, *P*=0.02), there were no differences in maternal/perinatal complications. Finally, making use of the placenta perfusion model, we established that both omeprazole and its S-isomer, esomeprazole, when maternally applied, reached the fetal compartment (fetal-to-maternal ratio’s 0.43–0.59), while only esomeprazole inhibited placental sFlt-1 release.

**Conclusions:**

Administration of omeprazole to women with confirmed preeclampsia does not alter their circulating levels of sFlt-1, PlGF, or ET-1, arguing against a role of this drug as a treatment for this syndrome.

**Novelty and Relevance**

**What Is New?**

- Preclinical studies suggest that proton pump inhibitors lower sFlt-1 (soluble Fms-like tyrosine kinase) and ET-1 (endothelin-1) in women with confirmed preeclampsia, thereby upregulating the non-sFlt-1-bound levels of placental growth factor.
- Yet, when treating women with confirmed preeclampsia with 40 mg omeprazole daily, no acute effect on either sFlt-1, ET-1, or placental growth factor was observed nor did this drug prolong pregnancy.
- Making use of the placenta perfusion model, omeprazole was observed to reach the fetal compartment.

**What Is Relevant?**

- Omeprazole does not alter the circulating levels of sFlt-1, ET-1, and placental growth factor in pregnancy, arguing against a role of this drug as a treatment for preeclampsia.

**Clinical/Pathophysiological Implications?**

- Given the lack of an acute effect of omeprazole in preeclamptic women, despite its earlier observed effect after longterm treatment and in preclinical studies, possibly lowering of sFlt-1 by proton pump inhibitors requires long-term treatment, started before the onset of preeclampsia.
- Thus, proton pump inhibitors should now be explored as preventive therapy rather than curative treatment.

Preeclampsia is a hypertensive syndrome unique to human pregnancy, with significant impact on maternal and fetal wellbeing worldwide.^1,2^ Approximately 5% of all pregnancies are affected by this syndrome, which is defined as the new onset of hypertension in the presence of maternal organ and/or uteroplacental dysfunction after 20 weeks’ gestation.^1,2^ Because there is no curative treatment for preeclampsia other than placental delivery, current management entails maternal administration of corticosteroids to accelerate fetal lung maturation, blood pressure control, and seizure prophylaxis while weighing the maternal risk of developing severe complications such as eclampsia, renal insufficiency, pulmonary edema and hemolysis, elevated liver enzymes, low platelets syndrome, against the danger of iatrogenic prematurity.^1^

The wide clinical variety and complexity of preeclampsia has tempered our ability to precisely understand the pathogenic mechanisms underlying this syndrome. Yet, a well-recognized phenomenon is that the placenta releases excessive amounts of sFlt-1 (soluble Fms-like tyrosine kinase-1) into the maternal circulation, which in turn binds to and reduces free PlGF (placental growth factor) and vascular endothelial growth factor. The ensuing highly antiangiogenic state subsequently triggers the release of the vasoconstrictor ET-1 (endothelin-1), further inducing the widespread maternal endothelial dysfunction typically associated with this syndrome.^3,4^ Ideally, mitigating the release of sFlt-1 and ET-1 or enhancing PlGF production should resolve the angiogenic imbalance, thereby delaying disease progression in women with preeclampsia. As such, current studies focus on novel therapies that could target these (anti-)angiogenic factors.^5^ Recently, proton pump inhibitors (PPIs) were shown to dose-dependently reduce sFlt-1 release in placental explants or trophoblast cells.^6^ PPIs are commonly prescribed for the treatment of gastric acid reflux and are considered safe for use in pregnancy. Following these observations, our group demonstrated that in gestational age (GA)-matched women with suspected or confirmed preeclampsia, PPI use was associated with lower sFlt-1 and ET-1 levels compared with women not using these drugs.^7^ Since most women (80%) in the former study were using omeprazole,^7^ we hypothesized that administering this PPI to women with confirmed preeclampsia could actively decrease the levels of circulating sFlt-1.

In the present study, we performed a randomized controlled trial to evaluate whether omeprazole treatment could alter the circulating and cord blood levels of sFlt-1, PlGF and CT-proET-1 (C-terminal pro-endothelin-1; a stable surrogate marker of ET-1^8^) in women with confirmed preeclampsia. In addition, making use of the ex vivo placenta perfusion model, we investigated the transplacental transfer of omeprazole (a racemic mixture of 2 optical isomers, R- and S-omeprazole, the latter being the active isomer) and its S-isomer, esomeprazole, and established whether they could effectively reduce placental perfusate levels of sFlt-1.

## Methods

All data and supporting materials have been provided within the published article.

### Study Design

This was a randomized controlled trial with individual randomization to the omeprazole group or non-omeprazole group according to GA. It was registered in the European Clinical Trials register, with protocol code number 64821. The trial was performed at 2 maternity units in Rotterdam, the Netherlands (Erasmus University Medical Center and Maasstad Hospital) between 2018 and 2021. All participants gave written informed consent to participate in the trial, which was approved by the Erasmus MC Research Ethics Committee (MEC-2018-071).

### Participants

Women were eligible to participate in the study if they had a confirmed diagnosis of preeclampsia, a GA between 20^+0^ and 34^+6^ weeks^+days^ on the day of randomization, a singleton pregnancy, were ≥18 years of age, and were able to give written informed consent. Women were not included in the trial if they were using any PPI at time of randomization, if they had a contraindication or hypersensitivity to PPI use, if they were using medication that could interact with PPI, if there was fetal death or distress at time of inclusion or if the clinicians expected delivery within the next 48 hours. The diagnostic criteria were based on the International Society of the Study of Hypertension in Pregnancy 2018 criteria, which defines preeclampsia as new-onset hypertension accompanied by proteinuria and/or maternal organ dysfunction and/or uteroplacental dysfunction at or after 20 weeks of gestation.^9^

### Sample Size Calculation

Based on the standardized effect size (1.0) and SD (0.93) calculated from the previous study conducted by Saleh et al,^7^ a sample size of 20 patients in both the intervention and control group was estimated. This would achieve 80% power to test the 67% decrease in sFlt-1 level reported in the study by Saleh et al,^7^ with a 2-sided alpha of 0.05.

### Randomization

Participants were allocated to receive omeprazole 40 mg, once daily or no omeprazole. Randomization was performed using an online, web-based sequence generator. Since the circulating biomarkers could significantly alter with advancing gestation, randomization blocks were stratified according to GA (strata 1 was <25 weeks, strata 2≥25 until <29 weeks, strata 3≥29 until <32 weeks, and strata 4≥33 until <35 weeks).

### Study Procedures

Women allocated in the omeprazole group received 30 capsules that were packaged for oral administration, with no special storage conditions. The omeprazole capsules were packaged and labeled by the pharmacy at the Erasmus Medical Center, which distributed the packs to the other participating center upon request. We advised that capsules should be taken once daily, preferably in the morning on an empty stomach, according to the pack’s instructions. Women in the omeprazole group that did not deliver 30 days after randomization would receive an additional omeprazole pack (containing 30 capsules). We recommended that treatment should be continued from enrollment until delivery. Compliance was monitored on a daily basis, since most women were admitted at the maternity units. After birth, all omeprazole packages were collected and counted by a research team member, to confirm compliance. In the non-omeprazole group, patients would not receive PPI, and those that developed gastric acid reflux during the trial, were advised to receive different drugs for this purpose. If despite these medications participants maintained gastric reflux requiring a PPI, they would be excluded.

At study enrollment, baseline data was entered on a web-based program by 2 independent researchers (R.I. Neuman and M.D. Baars).

Since the primary outcome of our study was the effect of omeprazole on maternal sFlt-1 production, we do not expect a placebo effect owing to the lack of blinding.

### Blood Collection and Biochemical Measurements

In both the non-omeprazole group and omeprazole group, serum and EDTA plasma was collected at baseline (day 0; before starting treatment with omeprazole), and day 1, 2, 4, 8 followed by twice weekly (during routine blood measurements) until delivery. The collected blood samples were stored after centrifugation, at −80 °C until analysis. Analysis of sFlt-1, PlGF, CT-proET-1 was performed at the end of the study. Serum sFlt-1 and PlGF were measured using an automated analyzer (Cobas e801 6000e, Roche Diagnostics, Almere, the Netherlands), while plasma CT-proET-1 levels were analyzed using a Thermo Fisher Kryptor Compact Plus (Thermo Fisher Scientific, BRAHMS GmbH, Henningsdorf, Germany).

### Outcomes

The primary outcome of the study was specified as the difference in sFlt-1, PlGF, and CT-proET-1 levels at day 4 after enrollment between the omeprazole group and the non-omeprazole group. Secondary outcomes comprised the difference in angiogenic markers 8 days after PPI administration, and the difference in longitudinal course or cord blood levels between the omeprazole and non-omeprazole group. Maternal and fetal/neonatal complications were assessed between groups. Maternal complications included eclampsia, pulmonary edema, placental abruption, subcapsular liver hematoma, cerebral hemorrhage/edema or infarction, visual disturbances, acute renal failure (absolute increase in the serum creatinine concentration of 0.3 mg/dL [26.4 µmol/L from baseline or >50% increase in serum creatinine; or oliguria with <0.5 mL/kg per hour for at least 6 hours) or postpartum hemorrhage (blood loss ≥1 L after delivery). Fetal/neonatal complications comprised of neonatal intensive care unit admission; small-for-gestational age infant (birth weight <10th percentile according to Dutch Perinatal Registration); endotracheal intubation; sepsis; respiratory distress syndrome; bronchopulmonary dysplasia (defined as chronic lung disease developing in preterm neonates treated with oxygen and positive-pressure ventilation, with radiographic signs of inflammation and scarring, in need of artificial ventilation 4 weeks post-partum and at 36 weeks postmenstrual age); necrotizing enterocolitis and fetal/neonatal death. All outcomes were recorded on the web-based trial database by 2 independent researchers (R.I. Neuman and M.D. Baars).

### Placenta Studies

#### Patients and Setting

Placentas of women who underwent an elective cesarean section with uncomplicated singleton pregnancies were collected immediately after delivery at the Erasmus Medical Center, Rotterdam, the Netherlands. Clinical characteristics were obtained from the patients’ electronic files. All women who donated their placenta gave approval through written informed consent. The study was exempted from approval by the local institutional Medical Ethics Committee according to the Dutch Medical Research with Human Subjects Law (MEC-2016-418 and MEC-2017-418).

#### Perfusion Experiments

The perfusion model used in the current study has been described by Hitzerd et al.^10^ Perfusion experiments were conducted in healthy, term placentas. Maternal and fetal perfusion media consisted of Krebs-Henseleit buffer at 37 °C, supplemented with heparin (final concentration; 2500 IU/L) and aerated with 95% O_2_ to 5% CO_2_. The fetal circulation (closed circuit; flow rate, 6 mL/minute) was established by cannulating the chorionic artery and corresponding vein of an intact cotyledon. Maternal circulation (closed circuit; flow rate 12 mL/minute) was created by placing 4 blunt cannulas in the intervillous space. At t=0, at a concentration of 10×C_max_, esomeprazole (50 µmol/L) or omeprazole (30 µmol/L), or no drug as a control were added to the maternal circulation. Samples of the maternal and fetal circulations were taken after 6 minutes and every 30 minutes until the end of the experiment (180 minutes) to determine the fetal-to-maternal (F/M) ratio and sFlt-1 concentration and were immediately stored at −80 °C. To establish good overlap between maternal and fetal circulations, antipyrine (100 mg/L) was added to the maternal buffer, while FITC-dextran (40 kDa, 36 mg/L) was added to the fetal circulation as a marker of integrity of the capillary bed. In order for an experiment to be successful, the F/M ratio of antipyrine had to be >0.75 and the maternal-to-fetal ratio of FITC-dextran <0.03 at t=180.

#### Liquid Chromatography-Mass Spectometry Analysis of S-Omeprazole and R-Omeprazole

The concentrations of S-omeprazole and R-omeprazole were measured in the perfusate by using a Waters Acquity UPC2-mass spectometry/mass spectometry system (Waters Corp, Milford, MA), equipped with a binary solvent pump including the CO_2_ and modifier pumps, a refrigerated autosampler, a convergence manager with back pressure regulator, and a column oven. Chiral separation was performed on an ACQUITY UPC^2^ Trefoil AMY1 column (100×2.1 mm, 2.5 µm), maintained at 20 °C. The mobile phase was composed of 80% CO_2_ and 20% ethanol/methanol (9/1, v/v) and was used at a flow rate of 1.5 mL/minute. A make-up flow consisted of the same composition and was set at 0.2 mL/minute. The back pressure regulator was set at 120 bar with a temperature of 70 °C. Total analysis time was 7 minutes. The auto sampler temperature was set at 5 °C. Perfusate samples were thawed and centrifuged, after which 50 µL was transferred to a microcentrifuge tube (1.5 mL). Protein precipitation was carried out by adding 1 mL of IS solved in methanol (ca. 1 mg/L). Next, the samples were vortex-mixed and centrifuged at 14 000 rpm for 5 minutes. The supernatant was transferred to vials, and a 5 µL aliquot was injected into the column, using a partial loop of 10 μL in needle overfill mode. Detection was performed on a Waters Xevo TQ-s micro mass spectrometry equipped with an electrospray ionization source, operated in positive ionization mode. The applied mass spectometry parameters were as follows: capillary voltage 3.5 kV, cone gas flow 1 L/hour, source block temperature 150 °C, and desolvation temperature 350 °C at a desolvation gas flow of 650 L/hour. Data acquisition was carried out using selected reaction monitoring. Ion transitions were m/z 346.18 to 198.13 for R/S-omeprazole and m/z 349.17 to 198.14 for the internal standard (S)-omeprazole-D3. A cone voltage of 20V and collision energy of 10V were used for both transitions. Raw data sets were processed by Masslynx software 4.1 and TargetLynx 4.1 (Waters Corp).

### Statistical Analysis

Data are reported as mean (±SD or SEM) or median (interquartile range) for continuous variables and as number (percentage) for categorical variables. The normality of continuous variables was assessed using the Shapiro-Wilk W test. For the comparison of continuous variables between 2 groups, an unpaired *t* test or Mann-Whitney *U* test (in case of non-normal distribution) was performed. For the comparison of categorical variables between 2 groups, Fisher exact or χ^2^ test was applied. We used linear mixed models to evaluate the longitudinal course of the angiogenic markers according to treatment group. To account for within-subject correlations, a random intercept for study subject was used. Time (days at which blood was taken) and GA at the first measurement were added as fixed effects. Baseline values (at day 0) were added as covariate. For the placenta studies, differences in sFlt-1 levels were studied using general linear model repeated measurements. A *P*<0.05 was considered statistically significant. Data were analyzed using IBM SPSS statistics (IBM Corporation, version 25) and R studio Statistical Software.

## Results

Between December 2018 and June 2021, a total of 87 women with preeclampsia were found eligible for inclusion, of whom 57 agreed to participate and were randomized. Of these, 50 were included in the outcome analysis of whom 26 were allocated to the non-omeprazole group and 24 to the omeprazole group (Figure S1). Baseline characteristics of all participants are displayed in Table 1. Mean maternal age was 31 years, and body mass index 26 kg/m^2^. Median GA at enrollment was 30 (28–31) weeks, which did not differ between the 2 groups (*P*=0.45)

**Table 1.**
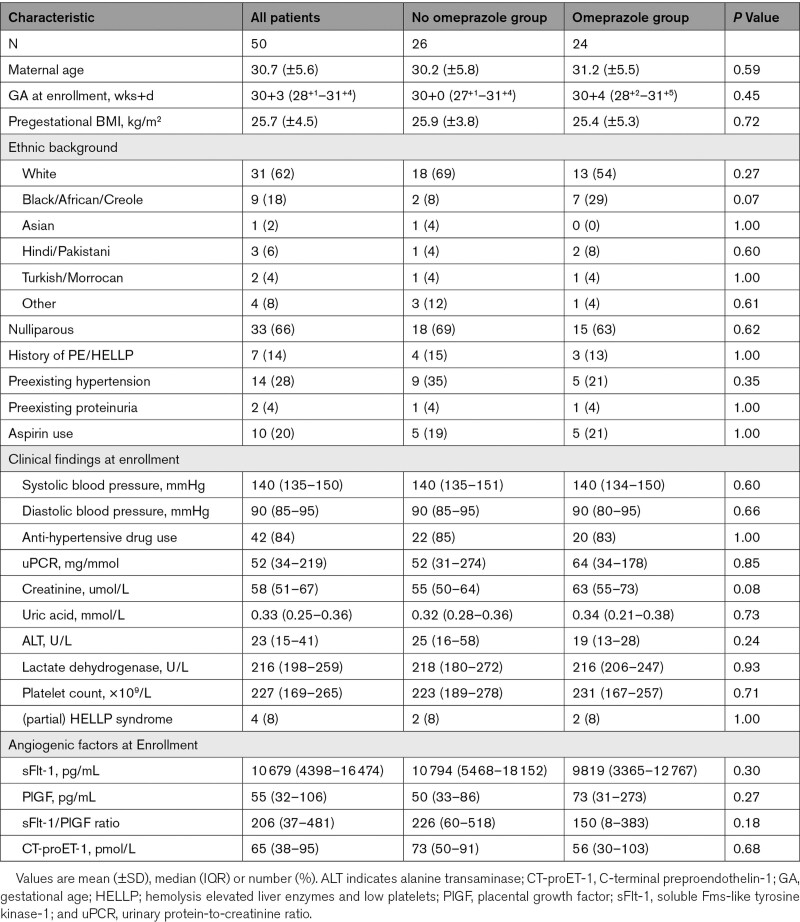
Baseline Characteristics of All 50 Participants at Enrollment

### Biomarker Levels 4 and 8 Days After Omeprazole Treatment

**Figure 1.**
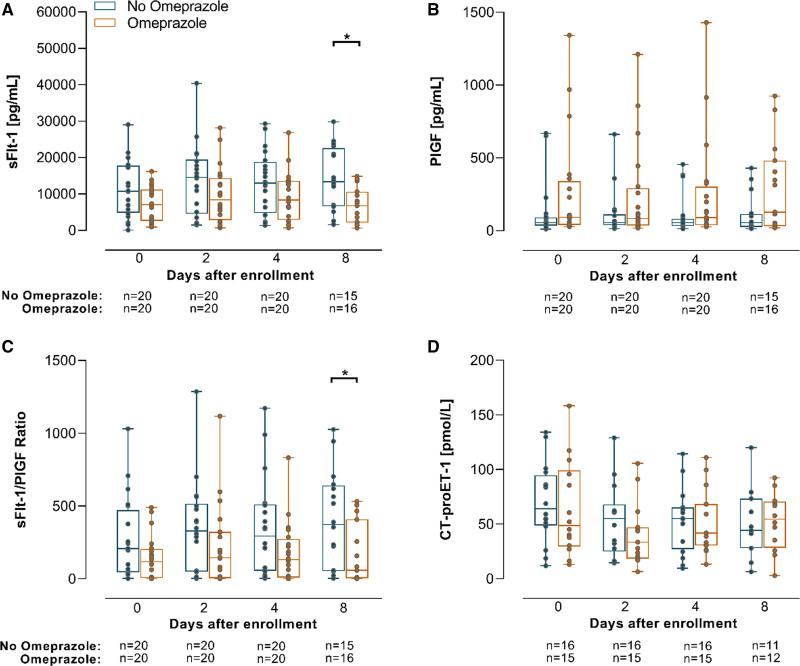
**Biomarkers after enrollment.** Box-and-whiskers plots showing circulating levels of sFlt-1 (soluble Fms-like tyrosine kinase-1) (**A**), PlGF (placental growth factor) (**B**), sFlt-1/PlGF ratio (**C**), and CT-proET-1 (C-terminal proendothelin-1) (**D**) in 40 singleton pregnancies according to days 0, 2, 4, and 8 after enrollment; * indicates *P*<0.05. Boxes show median and interquartile range, and whiskers are range.

Of the 50 women included for the outcome analysis, 10 were excluded from the primary outcome analysis either because they delivered within 4 days (n=9) or because biomarker measurements on day 4 were not performed according to protocol (n=1) (Figure S1). At baseline, levels of sFlt-1, PlGF, and sFlt-1/PlGF ratio were not significantly different between the omeprazole group (n=20) and the non-omeprazole group (n=20, median 7110 versus 10 743 pg/mL, *P*=0.11 for sFlt-1; 92 versus 55 pg/mL, *P*=0.14 for PlGF and 118 versus 208, *P*=0.08 for sFlt-1/PlGF ratio; Figure 1A through 1C). Of these women, those receiving omeprazole did not show lower sFlt-1 levels when measured 4 days after treatment compared with women not using omeprazole (8364 versus 13 017 pg/mL, *P*=0.14) nor did changes occur in the levels of PlGF (90 versus 55 pg/mL, *P*=0.14) or the sFlt-1/PlGF ratio (132 versus 294, *P*=0.06).

Thirty-one women remained pregnant after 8 days, of which those in the omeprazole group (n=16) displayed lower sFlt-1 levels (6800 versus 13 349 pg/mL, *P*=0.01) and a lower sFlt-1/PlGF ratio (59 versus 373, *P*=0.03) compared with the non-omeprazole group (n=15). However, baseline sFlt-1 levels in these 31 women were also significantly lower (*P*=0.01) in the omeprazole compared with the non-omeprazole group, and the same was true for the sFlt-1/PlGF ratio (*P*=0.02) (data not shown). CT-proET-1 measurements were only performed in 31 women, of which the values did not differ between groups at baseline, or at 4 or 8 days after enrollment (Figure 1D).

### Longitudinal Course of Biomarkers and Cord Blood Levels

**Figure 2.**
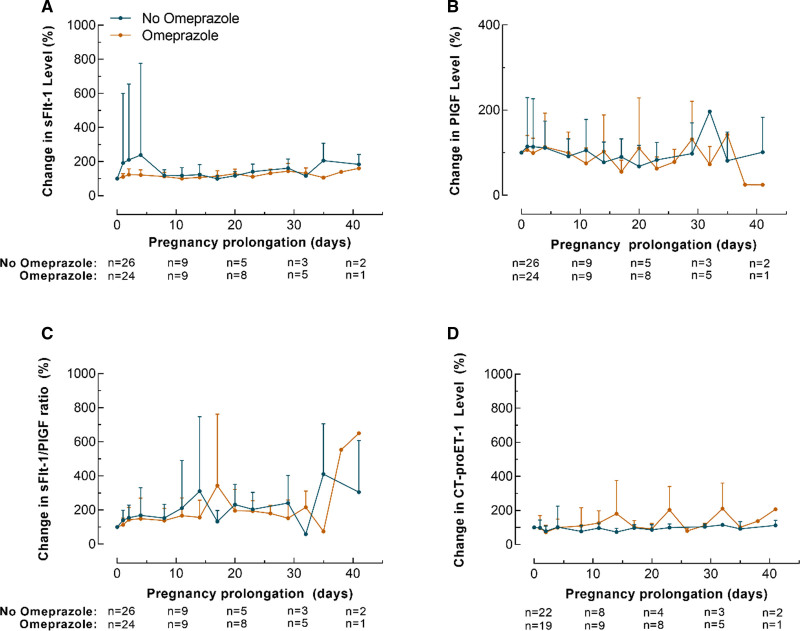
**Longitudinal changes in biomarkers. A**, sFlt-1 (soluble Fms-like tyrosine kinase-1), PlGF (placental growth factor) (**B**), sFlt-1/PlGF ratio (**C**), and CT-proET-1 (C-terminal proendothelin-1) (**D**) as percentages of baseline concentrations according to days of pregnancy prolongation. Values are expressed as median (interquartile range).

**Figure 3.**
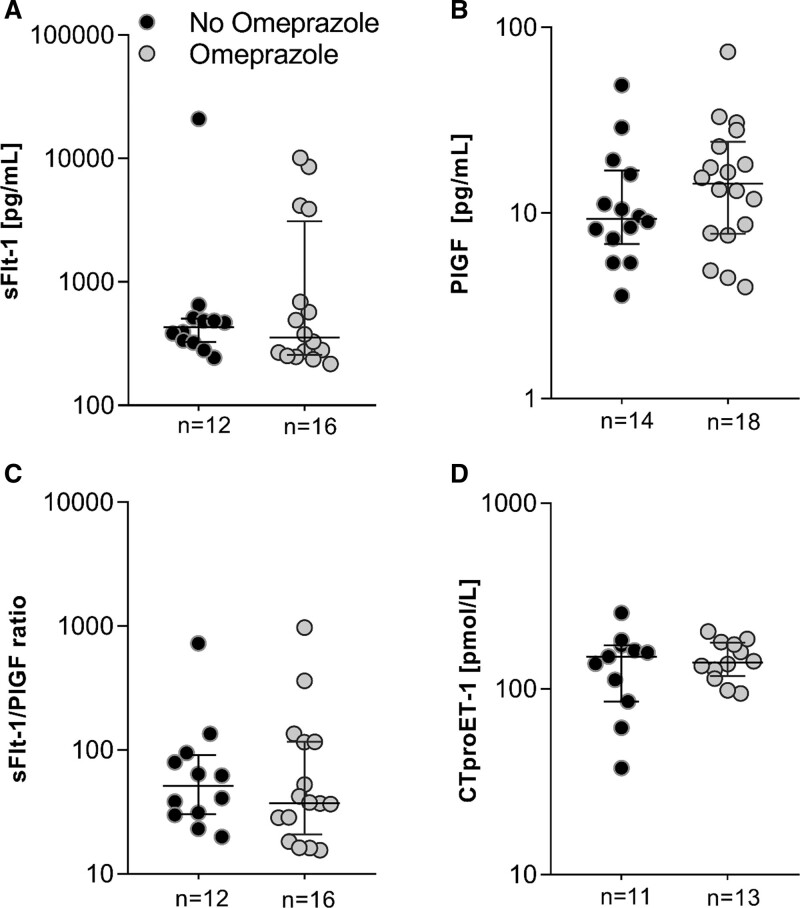
**Biomarkers in cord blood.** Cord blood levels of sFlt-1 (soluble Fms-like tyrosine kinase-1) (**A**), PlGF (placental growth factor) (**B**), sFlt-1/PlGF ratio (**C**), and CTproET-1 (C-terminal proendothelin-1) (**D**).

The longitudinal course differences in sFlt-1, PlGF, sFlt-1/PlGF ratio or CT-proET-1 levels were evaluated in all 50 women and could not be attributed to an effect of omeprazole treatment (fixed effect estimate of treatment group for sFlt-1, 281.76 [*P*=0.74]; PlGF, 36.04 [*P*=0.12]; sFlt-1/PlGF ratio, −37.6 [*P*=0.17] and for CT-proET-1, −3.8 [*P*=0.37]; Figure 2A through 2D). In addition, there were no differences in cord blood levels of these circulating factors between omeprazole group and non-omeprazole group (Figure 3A through 3D).

### Pregnancy Outcomes

Pregnancy outcomes were evaluated in all 50 included women. Mean GA at delivery was 33 weeks in the omeprazole group (n=24), compared with 32 weeks in the non-omeprazole group (n=26; *P*=0.36), as displayed in Table 2. The prolongation of pregnancy after enrollment of women receiving omeprazole was not different from that in women not using these drugs (median 15 versus 14 days, *P*=0.70). Adverse maternal outcomes did not occur more often in the non-omeprazole group in comparison to women receiving omeprazole (Table 2). With regard to neonatal complications, the proportion of adverse outcomes remained similar in both groups except for a higher percentage of neonates requiring endotracheal intubation in the non-omeprazole group compared with the omeprazole group (31% versus 4%, *P*=0.02).

**Table 2.**
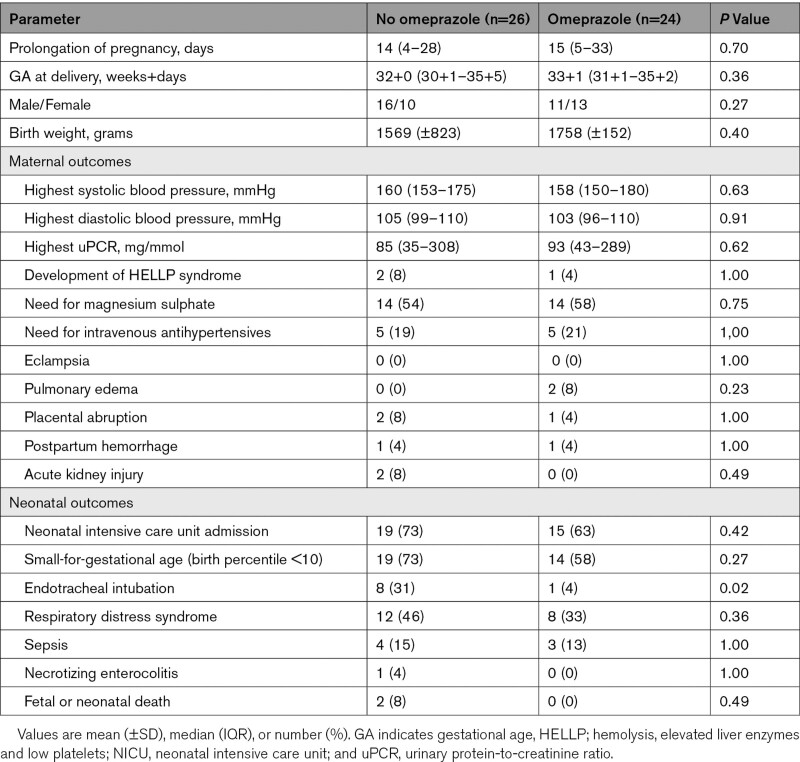
Pregnancy Outcomes According to Treatment Groups

### Placental Transfer of R-omeprazole and S-omeprazole

**Figure 4.**
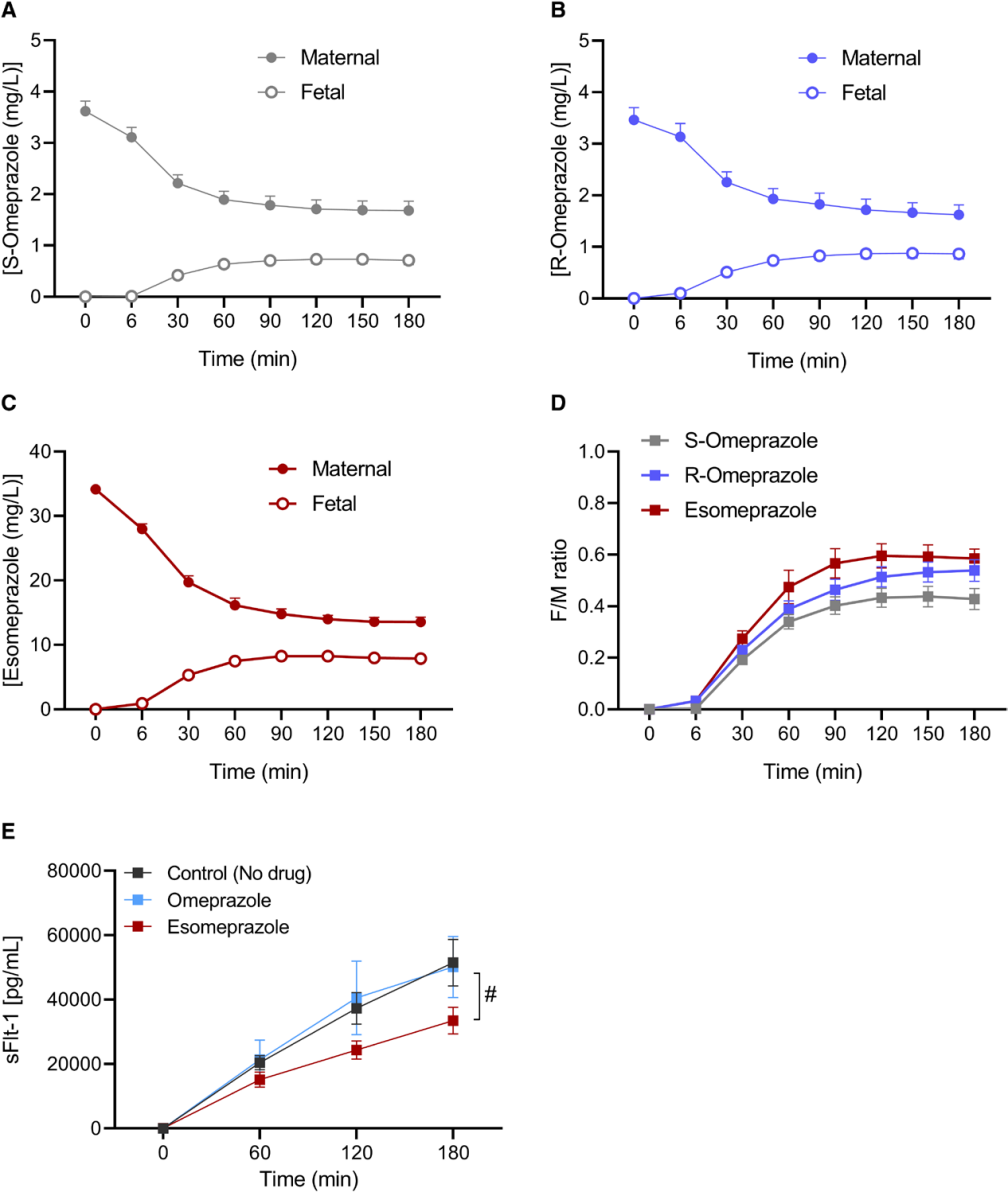
**Placental transfer of proton pump inhibitors. A**, S-omeprazole, R-omeprazole (**B**), and esomeprazole (**C**). **D**, The fetal-to-maternal (F/M) transfer ratios over time, and **E** shows the maternal effluent concentrations of sFlt-1 (soluble Fms-like tyrosine kinase-1) according to time (min) in placentas perfused with no drug (n=6), omeprazole (n=5), or esomeprazole (n=5). Values are depicted as mean (±SEM), # indicates *P*<0.05.

Of the 18 placentas of the women initially included in the perfusion study, 16 met the quality control criteria (success rate of 88%). Maternal characteristics and characteristics of the placentas and offspring are shown in Table S1. Figure 4 shows the transplacental transfer of omeprazole, divided into its S- and R-isomer (S-omeprazole and R-omeprazole), and the transfer of esomeprazole (=S-omeprazole). After 180 minutes of perfusion, the F/M ratio (±SEM) was 0.43±0.04 for the S-omeprazole, 0.54±0.04 for R-omeprazole, and 0.59±0.04 for esomeprazole. At this steady-state condition, there were no statistical differences in F/M ratio’s between these 3 groups (*P*=0.07).

### sFlt-1 Levels in Placental Perfusate

The maternal sFlt-1 release in esomeprazole-perfused placentas (n=5) was statistically significantly diminished in comparison to control placentas (*P*=0.01), as shown in Figure 4E. In contrast, maternal sFlt-1 perfusate concentrations in healthy placentas perfused with omeprazole (n=5) did not significantly differ from those placentas perfused without drugs (n=6, *P*=0.95).

## Discussion

In this randomized trial of women with confirmed preeclampsia, daily administration of 40 mg omeprazole did not significantly reduce the circulating levels of sFlt-1 and CT-proET-1, or increase the levels of PlGF after 4 or 8 days of treatment. Additionally, we observed no alterations in the longitudinal course of these angiogenic markers in maternal blood or in their cord blood levels, when comparing women receiving omeprazole compared with those not using this drug. When evaluating pregnancy outcomes, maternal complication rate was not different in the omeprazole group versus the non-omeprazole group, and the same was true for neonatal complications, except for a higher proportion of neonates requiring intubation in the non-omeprazole group. The present study is the first to evaluate the immediate consequences of omeprazole administration on circulating angiogenic marker levels in women with preeclampsia. Our results argue against a direct effect of omeprazole on sFlt-1 release as an explanation of our previous observation that sFlt-1 levels were 67% lower in 40 women with suspected or confirmed preeclampsia using PPIs compared with 80 gestational-age matched women not on this treatment.^7^ One reason for this discrepancy is that the median duration of PPI use was 29 days in the former,^7^ compared with just 14 days in the present study. Moreover, in a trial conducted by Cluver et al,^11^ daily administration of 40 mg esomeprazole for an average of 13 days also did not affect the serial course of sFlt-1 and PlGF in 59 women with preterm preeclampsia, in agreement with our findings. This suggests that, if PPIs could truly lower sFlt-1, this might only be observed after long-term treatment, requiring a start of this drug prior to the onset of preeclampsia. Here, we draw attention to the well-known magnesium-lowering effects of PPIs occurring after long-term treatment.^12^ Low magnesium levels, combined with the lower expression of the Mg^2+^-transporting transient receptor potential melastatin-subfamily member 7 in preeclamptic placentas, have been suggested to result in lower vascular endothelial growth factor expression.^13^ Although this concept provides a mechanistic basis for the therapeutic use of MgSO_4_ in preeclampsia, definite proof for this theory is lacking. If true, however, PPIs might exert both beneficial (lowering sFlt-1) and deleterious (lowering vascular endothelial growth factor) effects in preeclampsia. In fact, a population-based register cohort involving 157.720 nulliparous pregnant women, recently reported an increased risk of overall preeclampsia and preeclampsia at term of PPI use during any point of pregnancy, while PPI use recorded after 28 gestational weeks was associated with a reduced risk of preterm and early preeclampsia.^14^ Based on this, a unifying concept might be that to exert beneficial effects in preeclampsia, PPIs should be used over a defined period, in close proximity to disease onset, and most likely for >2 weeks. Interestingly, when we perfused healthy placentas with esomeprazole, maternal effluent levels of sFlt-1 were significantly lower in relation to controls. This was not observed for omeprazole. Similarly, Onda et al^6^ noted that esomeprazole and lansoprazole induced the highest dose-dependent sFlt-1 decrease in primary trophoblast and endothelial cells, whereas omeprazole exerted more modest reductions in sFlt-1. The simplest explanation of these findings is that, for a given concentration, esomeprazole contains double the amount of the active S-isomer. Furthermore, in the present study, the transplacental transfer of S-omeprazole alone (ie, esomeprazole) tended to be higher than its transfer in the presence of R-omeprazole (F/M ratio 0.59 versus 0.43; *P*=0.07), providing yet another reason for a higher tissue exposure to the active isomer, and thus a stronger effect, following esomeprazole exposure. Simultaneously, the in vivo half-life of the S-isomer is longer,^15^ but this is unlikely to be of relevance during a 3-hour placental perfusion period. Nevertheless, such acute effects of the S-isomer, if occurring at all, seem to be of limited relevance, since both esomeprazole^11^ and omeprazole (present study) were unable to diminish sFlt-1 release in an in vivo situation. Here, an important question remains why PPI-induced sFlt-1 reductions have been observed in preclinical studies, while we and others were not able to confirm these findings in clinical trials. Remarkably, this pattern has also been witnessed with other drugs such as pravastatin or sulfasalazine, which have shown significant sFlt-1-lowering properties in both placental tissues and animal models, although these actions were not apparent when examined in human patients.^16–20^ One possibility is that the doses applied in experimental models were much higher compared to the concentrations administered in clinical studies. Indeed, when the C_max_ equivalent of 40 mg esomeprazole (5 µmol/L) was applied in primary tissues, it did not induce a significant reduction in sFlt-1, in contrast with concentrations of 50 or 100 µmol/L.^6^ Increasing the dose (eg, to 80 mg) or applying these drugs intravenously might solve this problem, although there are limited data regarding pharmacokinetics and teratogenic risks for these concentrations. Indeed, since we observed a placental transfer of at least ≈40% to 50% to the fetal side, fetal drug levels may substantially increase when maternal blood concentrations are higher. In addition, it could be the case that the sFlt-1-diminishing effect of PPIs is simply insufficient to counteract the massive placental release when preeclampsia is already established. Therefore, we propose to explore PPIs as preventive therapy rather than curative treatment, for example, in combination with aspirin. In fact, a randomized-placebo controlled trial has already been initiated where the consequences of daily esomeprazole administration to women at high risk of preeclampsia will be assessed.^5^ We reported a substantially higher neonatal intubation rate in the nonomeprazole group compared with the omeprazole group. The nonsignificant higher baseline sFlt-1/PlGF ratio in the nonomeprazole group might partially explain higher disease severity and therefore more iatrogenic preterm birth in the latter group. However, the proportion of neonates admitted to the neonatal intensive care unit or presenting with other complications remained similar between groups, arguing against this hypothesis. Moreover, in our former analysis evaluating PPI users, a nonsignificant trend toward higher intubation rate was similarly observed in the non-PPI group compared with the PPI group (15% versus 5%, *P*=0.07).^7^ On the other hand, in the trial by Cluver et al,^11^ where a much larger population was evaluated, a difference in intubation rate between esomeprazole and placebo group was not found. Hence, caution is granted when interpreting these findings, since our study is underpowered for these outcomes.

## Perspectives

Our study has several strengths. Importantly, our population accurately reflects the clinical characteristics of women with preterm preeclampsia, of which >70% had a sFlt-1/PlGF ratio ≥85. In addition, we were able to closely monitor all participants, leading to a high compliance rate in our trial. Since most women already delivered within 1 to 2 weeks after enrollment, a limitation of our study was the low number of women assessed for the serial course of the biomarkers between the omeprazole and non-omeprazole group. Nevertheless, this was a secondary outcome, similar to the maternal/neonatal adverse outcomes, whereas our power calculation was solely based on detecting a difference in sFlt-1. In summary, our findings argue against a role for PPIs as potential treatment for preeclampsia, since daily administration of this drug was unable to improve the maternal circulating profile of sFlt-1, PlGF, and ET-1 in women with established preeclampsia.

## Article Information

### Acknowledgments

We thank Roche Diagnostics for providing the kits to measure sFlt-1 (soluble Fms-like tyrosine kinase-1) and PlGF (placental growth factor), and Thermo Fisher Scientific for providing the kit to measure CT-proET-1 (C-terminal proendothelin-1).

### Sources of Funding

R.I. Neuman and A.H. van den Meiracker are supported by the Dutch Foundation Lijf en Leven.

### Disclosures

None.

## Supplementary Material


