# Supplementary material for: Omeprazole Administration in Preterm Preeclampsia: a Randomized Controlled Trial to Study Its Effect on sFlt-1 (Soluble Fms-Like Tyrosine Kinase-1), PlGF (Placental Growth Factor), and ET-1 (Endothelin-1)
Source: Hypertension. 2022 Apr 28;79(6):1297–307. doi: 10.1161/HYPERTENSIONAHA.122.19070 (PMC9093236; doi:10.1161/HYPERTENSIONAHA.122.19070)

Omeprazole Administration in Preterm Preeclampsia: A Randomized Controlled Trial to Study its Effect on Soluble Fms-like Tyrosine Kinase-1, Placental Growth Factor and Endothelin-1.

Rugina I. Neuman[1,2], Milan D. Baars[2], Langeza Saleh[1,2], Michelle Broekhuizen[1,3], Daan Nieboer[4], Jérôme Cornette[2], Sam Schoenmakers[2], Michel Verhoeven[5], Birgit C.P. Koch[5], Henk Russcher[6], Sjoerd A.A. van den Berg[6,7], Anton H. van den Meiracker[1], Willy Visser[1,2], A.H. Jan Danser[1]

[1]Department of Internal Medicine, Division of Pharmacology and Vascular Medicine;

[2]Department of Obstetrics and Gynecology, Division of Obstetrics and Fetal Medicine;

[3]Department of Pediatrics, Division of Neonatology;

[4]Department of Biochemical Statistics;

[5]Department of Pharmacy;

[6]Department of Clinical Chemistry;

[7]Department of Internal Medicine, Division of Endocrinology,

Erasmus MC University Medical Center, Rotterdam, The Netherlands

Corresponding author:

Prof. dr. A.H.J. Danser

Department of Internal Medicine, Room Ee-1418B

Erasmus Medical Centre

P.O. Box 2040, 3000 CA Rotterdam, The Netherlands

Telephone number: 0031107043540

E-mail: a.danser@erasmusmc.nl

SUPPLEMENTAL MATERIAL

Table S1. Characteristics of the patients whose Placentas were Perfused with No drug, Omeprazole or Esomeprazole.

**Characteristic**	**No drug (n=6)**	**Omeprazole (n=5)**	**Esomeprazole (n=5)**
Maternal age, years	35 (34 - 36)	32 (28 - 33)	37 (35 - 38)
Parity, n	1 (1- 1)	1 (0 - 1)	1 (1 - 1)
Caucasian Background, n	4	2	3
Body Mass index, kg/m^2	26 (24 - 30)	24 (22 - 25)	23 (22 - 25)
Highest DBP, mmHg	85 (73 - 88)	80 (75 - 85)	87 (75 - 89)
Gestational age at delivery, weeks	39 (38 - 39)	39 (38 - 39)	39 (39 - 39)
Birth weight, grams	3502 (3377 - 3708)	3300 (2960 - 3555)	3738 (3685 - 3825)
Birth weight percentile, grams	61 (55 - 77)	51 (28 - 71)	84 (82 - 90)
Placental weight, grams	687 (653 - 743)	660 (494 - 680)	695 (645 - 727)
Fetal sex (Female/Male)	4/2	5/0	5/0

Values are median (IQR) or number (%). DBP indicates diastolic blood pressure and GA, gestational age,

Table S2A. Baseline characteristics of 40 participants only included in the primary outcome.

Characteristic	All patients	No Omeprazole group	Omeprazole group	P-Value
N	40	20	20	
Maternal age	31.3 (±5.8)	31.1 (±6.2)	31.5 (±5.6)	0.72
GA at enrollment, weeks+days	30+2 [28+1 - 31+6]	29+1 [27+0 - 31+5]	30+4 [28+2 - 31+6]	0.30
Pregestational BMI, kg/m^2	26.4 (23.8 - 28.4)	26.5 (±3.7)	25.1 (±5.3)	0.22
**Ethnic Background**				
Caucasian/White	23	13 (65)	10 (50)	NS
Black/African/Creole	9	2 (10)	7 (35)	NS
Asian	1	1 (5)	0 (0)	NS
Hindi/Pakistani	3	1 (5)	2 (10)	NS
Turkish/Morrocan	1	1 (5)	0 (0)	NS
Other	3	2 (10)	1 (5)	NS
Nulliparous	26	14 (70)	12 (60)	0.51
History of Preeclampsia/HELLP	6	4 (20)	2 (10)	0.66
Pre-existing hypertension	12	7 (35)	5 (25)	0.73
Pre-existing proteinuria	2	1 (5)	1 (5)	1.00
**Clinical findings at enrollment**				
Systolic blood pressure, mmHg	140 (135 - 150)	140 (136 - 152)	140 (134 - 150)	0.48
Diastolic blood pressure, mmHg	90 (84 - 95)	90 (84 - 95)	90 (81 - 95)	0.98
Anti-hypertensive drug use	33	17 (52)	16 (49)	1.00
uPCR, mg/mmol	54 (35 - 183)	52 (30 - 198)	69 (39 - 178)	0.51
Creatinine, umol/L	58 (51 - 64)	55 (51 - 64)	61 (53 - 68)	0.35
Uric acid, mmol/L	0.39 (0.23 - 0.35)	0.30 (0.29 - 0.36)	0.26 (0.20 - 0.35)	0.22
ALT, U/L	24 (15 - 42)	25 (16 - 53)	21 (12 - 33)	0.38
Lactate dehydrogenase, U/L	213 (187 - 258)	203 (178 - 267)	216 (200 - 247)	0.86
Platelet count, x10^9/L	227 (169 - 264)	219 (197 - 266)	236 (157 - 263)	0.80
(partial) HELLP syndrome	3 (8)	1 (5)	2 (10)	1.00
**Angiogenic Factors at Enrollment**				
sFlt-1, pg/mL	9819 (3791 - 12767)	10743 (4884 - 17834)	7110 (2633 - 11227)	0.11
PlGF, pg/mL	61 (39 - 199)	55 (36 - 92)	92 (40 - 340)	0.14
sFlt-1/PlGF ratio	160 (17 -252)	208 (46 - 471)	118 (7 - 206)	0.08
CT-proET-1, pmol/L	64 (44 - 98)	59 (50 - 91)	86 (28 - 113)	0.79

Values are mean (±SD), median (IQR) or number (%). GA indicates gestational age, HELLP; hemolysis, elevated liver enzymes and low platelets; uPCR, urinary protein-to-creatinin ratio; ALT, alanine transaminase; sFlt-1, soluble Fms-like tyrosine kinase-1; PlGF, placental growth factor; CT-proET-1, C-terminal preproendothelin-1.

Table S2B. Pregnancy outcomes according to treatment groups in the 40 participants included for primary outcome analysis.

Characteristic	No Omeprazole (n = 20)	Omeprazole (n = 20)	P-Value
Time to delivery, days	22 (8 - 34)	19 (8 - 39)	0.86
GA at delivery, weeks+days	32+5 (30 + 1 - 37 + 0)	33+4 (32 +1 - 36+6)	0.38
Male/Female	12/8 (60/40)	10/10 (50/50)	0.75
Birth weight, grams	1669 (±892)	1894 (±744)	0.27
**Maternal Outcomes**			
Highest systolic blood pressure, mmHg	165 (156 - 177)	158 (150 - 180)	0.38
Highest diastolic blood pressure, mmHg	105 (100 -110)	103 (96 - 110)	0.74
Highest urinary protein-to-creatinin ratio, mg/mmol	85 (38 - 252)	71 (43 - 286)	0.76
Development of HELLP syndrome	2 (10)	1(5)	1.00
Need for magnesium sulphate during trial	9 (45)	10 (50)	0.75
Need for intravenous antihypertensives during trial	3 (15)	4 (21)	0.69
Eclampsia	0(0)	0(0)	1.00
Pulmonary edema	0 (0)	1 (5)	1.00
Placental abruption	1 (5)	0 (0)	1.00
Postpartum hemorrhage	1 (5)	1 (5)	1.00
Acute kidney injury	2 (10)	0 (0)	0.49
**Neonatal outcomes**			
Neonatal Intensive Care Unit admission	13 (65)	11 (55)	0.75
Small-for-gestationale age infant (percentile <10)	14 (70)	10 (50)	0.19
Endotracheal intubation	6 (30)	1 (5)	0.09
Respiratory distress syndrome	8 (40)	6 (30)	0.51
Sepsis	3 (15)	3 (15)	1.00
Necrotising enterocolitis	1 (5)	0 (0)	1.00
Fetal or neonatal death	1 (5)	0 (0)	1.00

Values are mean (±SD), median (IQR) or number (%). GA indicates gestational age, HELLP; hemolysis, elevated liver enzymes and low platelets; uPCR, urinary protein-to-creatinine ratio; NICU, neonatal intensive care unit.

Figure S1. Flow diagram.

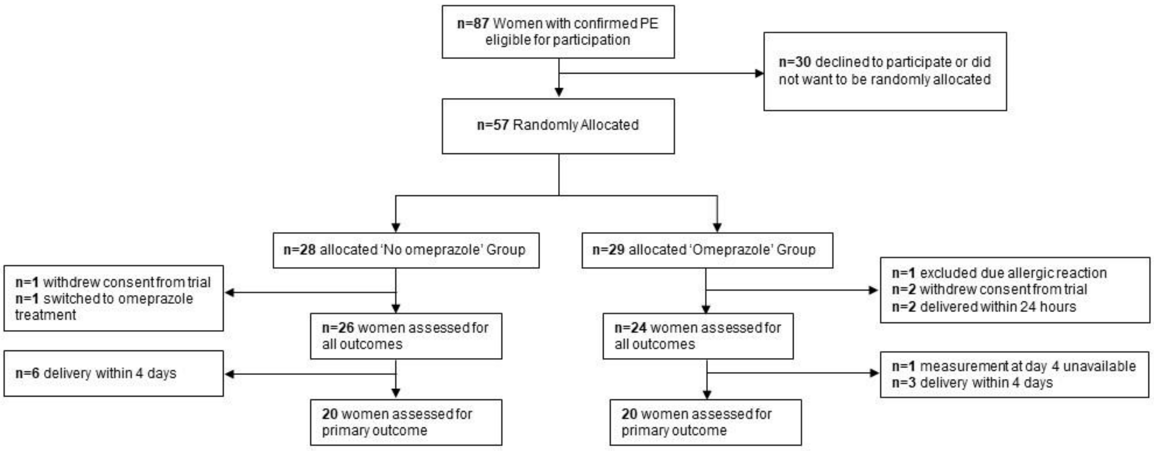

Supplement: Supplementary file 1 [file hyp-79-1297-s001.pdf]
